# The role of β‐barrels 1 and 2 in the enzymatic activity of factor XIII A‐subunit

**DOI:** 10.1111/jth.14128

**Published:** 2018-05-27

**Authors:** E. L. Hethershaw, P. J. Adamson, K. A. Smith, W. N. Goldsberry, R. J. Pease, S. E. Radford, P. J. Grant, R. A. S. Ariëns, M. C. Maurer, H. Philippou

**Affiliations:** ^1^ Discovery and Translational Science Department Leeds Institute for Cardiovascular and Metabolic Medicine Faculty of Medicine and Health University of Leeds Leeds UK; ^2^ Chemistry Department University of Louisville Louisville KY USA; ^3^ Astbury Centre for Structural Molecular Biology School of Molecular and Cellular Biology University of Leeds Leeds UK

**Keywords:** catalytic domain, enzyme activation, factor XIII, protein conformation, transglutaminases

## Abstract

**Essentials**

- The roles of β‐barrels 1 and 2 in factor XIII (FXIII) are currently unknown.
- FXIII truncations lacking β‐barrel 2, both β‐barrels, or full length FXIII, were made.
- Removing β‐barrel 2 caused total loss of activity, removing both β‐barrels returned 30% activity.
- β‐barrel 2 is necessary for exposure of the active site cysteine during activation.

**Summary:**

**Background**

Factor XIII is composed of an activation peptide segment, a β‐sandwich domain, a catalytic core, and, finally, β‐barrels 1 and 2. FXIII is activated following cleavage of its A‐subunits by thrombin. The resultant transglutaminase activity leads to increased resistance of fibrin clots to fibrinolysis.

**Objectives**

To assess the functional roles of β‐barrels 1 and 2 in FXIII, we expressed and characterized the full‐length FXIII A‐subunit (FXIII‐A) and variants truncated to residue 628 (truncated to β‐barrel 1 [TB1]), residue 515 (truncated to catalytic core [TCC]), and residue 184 (truncated to β‐sandwich).

**Methods**

Proteins were analyzed by gel electrophoresis, circular dichroism, fluorometric assays, and colorimetric activity assays, clot structure was analyzed by turbidity measurements and confocal microscopy, and clot formation was analyzed with a Chandler loop system.

**Results and Conclusions**

Circular dichroism spectroscopy and tryptophan fluorometry indicated that full‐length FXIII‐A and the truncation variants TCC and TB1 retain their secondary and tertiary structure. Removal of β‐barrel 2 (TB1) resulted in total loss of transglutaminase activity, whereas the additional removal of β‐barrel 1 (TCC) restored enzymatic activity to ~ 30% of that of full‐length FXIII‐A. These activity trends were observed with physiological substrates and smaller model substrates. Our data suggest that the β‐barrel 1 domain protects the active site cysteine in the FXIII protransglutaminase, whereas the β‐barrel 2 domain is necessary for exposure of the active site cysteine during activation. This study demonstrates the importance of individual β‐barrel domains in modulating access to the FXIII active site region.

## Introduction

**Figure 1:**
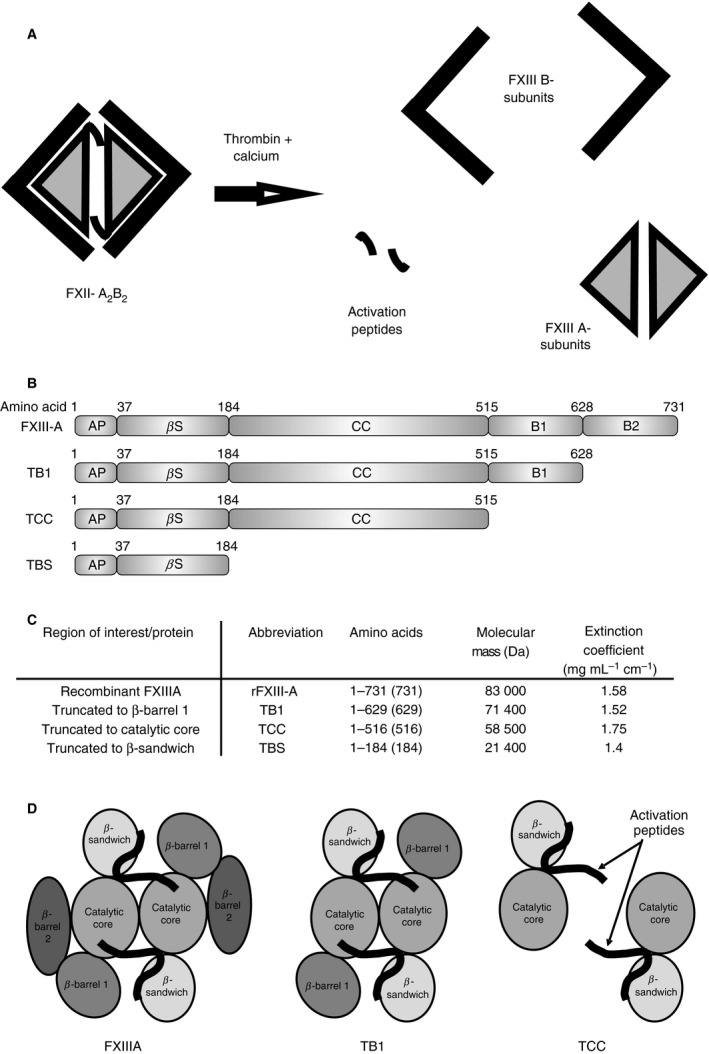
Activation of FXIII and Factor XIIIA fragments. Schematic of activation of FXIII by thrombin and calcium (A). Recombinant full length FXIII A subunit, TB1 (truncation to residue 628 lacking barrel 2), TCC (truncation to residue 513 lacking barrels 1 and 2), and TBS (truncation to residue 184 lacking catalytic core, and barrels 1 and 2) were successfully produced (B & C). Schematic of FXIIIA, TB1, and TCC (D). FXIIIA, full length FXIII A subunit; TB1, full length FXIII A subunit lacking barrel 2; TBS, full length FXIII A subunit lacking barrels 1 and 2, and catalytic core; TCC, full length FXIII A subunit lacking barrels 1 and 2.

In the final step of the blood coagulation cascade, fibrin monomers polymerize to generate a fibrin clot. Activated factor XIII (FXIIIa) catalyzes the formation of ε‐(γ‐glutamyl)lysine covalent bonds between glutamine and lysine residues of adjacent fibrin molecules 1. FXIIIa is also capable of crosslinking other substrates into the fibrin clot network whose functions include inhibition of fibrinolysis (e.g. α_2_‐antiplasmin 2, 3), increased thrombin generation at the clot surface (e.g. FV 4, 5), and platelet adhesion to the clot (e.g. collagen 6, 7). FXIII is a 320‐kDa heterologous tetramer comprising two A‐subunits, which contain the active site of the enzyme 8, and two B‐subunits, which stabilize the hydrophobic A‐subunits in the plasma 9, 10 (Fig. 1A). The A‐subunits are folded into four distinct domains, from N‐terminus to C‐terminus: the activation peptide, β‐sandwich, catalytic core (containing the active site), β‐barrel 1 and β‐barrel 2 domains 8 (Fig. 1B). The dimer folds with the β‐barrel domains arranged around the outside of the protein structure. Thrombin cleaves the activation peptide from the N‐terminus of each A‐subunit monomer and, in the presence of calcium, the B‐subunits of FXIII dissociate from the A‐subunits, exposing the active sites of the A‐subunits in the catalytic core to substrates 11, 12, 13.

Previous studies have shown that FXIII‐A_2_ undergoes conformational changes upon thrombin cleavage of the activation peptide 12, 14 and in the presence of calcium 15, 16, 17. It is also known that fibrin enhances thrombin cleavage of the activation peptide and contributes a binding surface for FXIII 18, 19, 20, 21, 22. Fibrin thus both aids in FXIII activation and itself serves as a transglutaminase substrate. Although the precise changes that occur to FXIII are not fully understood, there is strong evidence that the β‐sandwich, β‐barrel 1 and β‐barrel 2 all play a role in the conformation of the catalytic core 13, 23, 24. Using a recombinant FXIII‐A_2_ truncation variant that lacks either β‐barrel 2 or both β‐barrel domains, we were able to show that β‐barrels 1 and 2 are crucial for full enzymatic activity of the protein.

## Materials and methods

### Production of recombinant FXIII A‐subunit (FXIII‐A) and truncations

Recombinant FXIII‐A was expressed in *Escherichia coli* and purified as described previously 25. Further experimental details of the expression of the truncation variants are provided in Data S1.

### SDS‐PAGE and densitometry analysis

Recombinant proteins were subjected to SDS‐PAGE gel analysis under reducing conditions in precast 4–12% Bis‐Tris gels (Life Technologies, Paisley, UK). Gels were stained with Coomassie blue, and subjected to densitometry analysis with ID 3.1 Image software supplied with the Kodak IS2000R Imager (Eastman Kodak Company, New Haven, CT, USA).

### Fluorometry

Fluorescence emission spectra of recombinant full‐length FXIII‐A and variants truncated to residue 628 (truncated to β‐barrel 1 [TB1]) and residue 515 (truncated to catalytic core [TCC]) at 1.2 μm in 10 mm 3‐(*N*‐morpholino)propanesulfonic acid (MOPS), pH 7.4, were collected by use of a Varioskan Flash fluorescence plate reader (Thermo Fisher Scientific, Waltham, MA, USA) at 25 °C. Tryptophan residues of samples were excited at 280 nm, and emission spectra were collected in triplicate from 300 nm to 400 nm in 1‐nm steps. Blanks in the absence of proteins were measured in triplicate and subtracted from the protein spectra. Precise sample concentrations were determined by quantitative amino acid analysis and spectra‐compensated accordingly.

### Circular dichroism (CD)

Purified full‐length recombinant FXIII‐A and truncations TB1 and TCC were dialyzed into 10 mm MOPS, pH 7.4 and the concentrations were adjusted to 0.14 mg mL^−1^. Far‐UV CD spectra were recorded in a Jasco J‐715 Spectropolarimeter (Jasco, Great Dunmow, UK) at 21 °C at 0.2‐nm intervals over a wavelength range from 190 nm to 300 nm, in a 1‐cm quartz Suprasil cuvette (Hellma, Southend on Sea, UK). Three scans of each spectra were averaged, baseline‐subtracted against buffer, and corrected to equal molar concentrations. Baseline‐subtracted spectra were zeroed between 263 nm and 270 nm. Spectra were smoothed with the Savitsky–Golay algorithm in cdtool software 26. Sample concentrations were determined by quantitative amino acid analysis, and spectra were converted to mean residue ellipticity (degrees cm^2^ dmol^−1^ residue^−1^).

### Determination of transglutaminase activity by pentylamine incorporation

The activities of recombinant full‐length FXIII‐A, TB1 and TCC and a variant truncated to residue 184 (truncated to β‐sandwich [TBS]) were determined with a pentylamine incorporation assay as described previously 27. Briefly, microtiter plates were coated with either 100 μg mL^−1^ casein (Sigma Aldrich, Gillingham, Dorset, UK) at 4 °C overnight or 40 μg mL^−1^ human fibrinogen (Enzyme Research Laboratories, Swansea, UK) at 37°C for 1 h. After blocking with 1% bovine serum albumin (BSA), plates were incubated with triplicates of 3.5 nm recombinant FXIII‐A sample, 0.27 μm 5‐(biotinamido)pentylamine (Thermo Fisher Scientific, Rockford, IL, USA), 1 U mL^−1^ human thrombin (Calbiochem, Merck, Darmstadt, Germany), 100 μm dithiothreitol (DTT), and 1 mm CaCl_2_. Incorporation of 5‐(biotinamido)pentylamine was stopped with 133 mm EDTA over a time course of 25 min or 120 min for the fibrinogen or casein substrate, respectively. Crosslinking of the 5‐(biotinamido)pentylamine into the fibrin by recombinant FXIII was detected by the use of streptavidin–alkaline phosphatase (Life Technologies) and *p*‐nitrophenyl phosphate (Sigma Aldrich). Plates were measured at an OD of 405 nm in an ELx808 multiwell plate reader (BioTek, Winooski, VT, USA).

### Determination of protein activity by α_2_‐antiplasmin incorporation

The activities of recombinant FXIII‐A, TB1, TCC and TBS were also assayed by α_2_‐antiplasmin incorporation, based on a method previously described 28. Briefly, microtiter plates were coated with 40 μg mL^−1^ human fibrinogen (Enzyme Research Laboratories) at 37 °C for 1 h. After blocking with 1% BSA, plates were incubated with 1 U mL^−1^ human thrombin (Calbiochem) and 5 mm CaCl_2_ to convert fibrinogen to fibrin, and then treated in triplicate with 3.5 nm recombinant FXIII‐A sample, 10 μg mL^−1^ α_2_‐antiplasmin (Calbiochem), 1 U mL^−1^ human thrombin, 0.1 mm DTT, and 1 mm CaCl_2_. Incorporation of α_2_‐antiplasmin was stopped with 133 mm EDTA over a time course of 50 min. Crosslinking of the α_2_‐antiplasmin into the fibrin by recombinant FXIII was detected by the use of goat anti‐human α_2_‐antiplasmin antibody with a horseradish peroxidase conjugate (Enzyme Research Laboratories) and *o*‐phenylenediamine (OPD) (Dako, Ely, UK). Plates were measured at 490 nm in a multiwell plate reader (ELx808; BioTek).

### Determination of transglutaminase activity with a Q‐containing substrate peptide

A matrix‐assisted laser desorption ionization time‐of‐flight (MALDI‐TOF) mass spectrometry (MS) assay was employed to monitor FXIIIa‐catalyzed depletion of a model peptide into its crosslinked product 29. K9 peptide (LGPGQSKVIG) served as the glutamine‐containing substrate, and glycine ethyl ester (GEE) as a lysine mimic. Each reaction mix contained 220 nm FXIII‐A (full length, TB1, or TCC), 3 mm CaCl_2_, and 5 mm GEE, all in Tris‐acetate buffer. Bovine thrombin (7 U mL^−1^; Sigma Aldrich) was added and incubated at 37 °C for 5 min. K9 peptide (400 μm; Peptides International, Louisville, KY, USA) was then added. After 5, 10 and 30 min, aliquots were quenched with 5 mm EDTA. Samples were later run on a MALDI‐TOF mass spectrometer, and the percentage of reactant left at each time was calculated as follows:

$$\frac{\sum \text{Reactant Peak Height}*100}{\sum \text{Reactant Peak Height}+\sum \text{Product Peak Height}}$$

Assays were performed in triplicate, and standard deviations were calculated.

### Depletion of FXIII from fibrinogen

FXIII‐depleted fibrinogen was purified from human fibrinogen (Enzyme Research Laboratories) by ammonium sulfate precipitation, as previously described 30.

### Turbidity

Polymerization of fibrinogen in the presence of full‐length or truncated recombinant FXIII‐A was measured with a microtiter plate turbidity assay as previously described 31. Clots were formed in triplicate with 1 mg mL^−1^ FXIII‐depleted fibrinogen, 65 nm recombinant wild‐type FXIII‐A or truncated FXIII‐A, 0.125 U mL^−1^ human thrombin (Calbiochem), and 5 mm CaCl_2_. The increase in turbidity was continually monitored at 340 nm every 12 s in a multiwell plate reader (ELx808; BioTek) for 60 min at 37 °C.

### Confocal microscopy

Fibrinolysis rates of fibrin clots formed in the presence of full‐length or truncated recombinant FXIII‐A were measured by the use of non‐fluorescence confocal microscopy as previously described 32. Clots were formed in triplicate, with the same concentrations of reactants as used for the turbidity experiments. Lysis was then initiated with 280 μg mL^−1^ plasminogen (Enzyme Research Laboratories) and 1 μg mL^−1^ tissue‐type plasminogen activator (t‐PA) (Technoclone, Vienna, Austria). The clot was visualized under low magnification every 20 s with a Leica TCS SP‐2 laser scanning 1072 confocal microscope (Leica Microsystems, Heidelberg, Germany), and the time taken for the lysis front to migrate from a fixed point was measured. The lysis front velocity was determined and used to calculate the mean overall lysis rate in μm s^−1^.

### Labeling fibrinogen with Alexa Fluor 488

FXIII‐depleted fibrinogen was labeled with the fluorophore Alexa Fluor 488 (Life Technologies). One milligram of the fluorophore was mixed with 24 mg of FXIII‐depleted fibrinogen, and incubated on a roller at room temperature for 60 min. The unreacted fluorophore was then removed by exhaustive dialysis into Tris‐buffered saline (pH 7.4). The degree of labeling was determined to be three or four molecules of dye per one molecule of fibrinogen, according to the manufacturer's protocol.

### Chandler loop

Fibrinolysis rates of fibrin clots formed in the presence of FXIII‐A were also measured under flow with a Chandler loop system as previously described 32, 33. Clots were formed by the use of FXIII‐depleted fibrinogen containing 5% Alexa Fluor 488‐conjugated FXIII‐depleted fibrinogen. The same concentrations of reactants as used for the turbidity experiments were used. After 2 h, clots were retained within the tubing and washed with Tris‐buffered saline. Lysis was initiated with 28 μg mL^−1^ plasminogen (Enzyme Research Laboratories) and 0.1 μg mL^−1^ t‐PA (Technoclone, Vienna, Austria).

### Crosslinking of fibrin

Clots were formed at 37 °C by the use of 400 μg mL^−1^ FXIII‐depleted fibrinogen, 26 nm recombinant wild‐type FXIII‐A or truncated FXIII‐A, 0.125 U mL^−1^ human thrombin (Calbiochem), and 5 mm CaCl_2_. The reaction was stopped after 0, 5, 30, 60, 90 and 180 min by the addition of reducing sample buffer (Life Technologies) and heating the samples for 10 min at 95 °C. Samples were run and visualized as described above.

### Statistical analysis

All statistical analyses were performed with pasw 21.0 (SPSS, Chicago, IL, USA). Data are expressed as mean and standard error of the mean. One‐way anova with Bonferroni *post hoc* analysis was used, and *P*‐values of < 0.05 were considered to be statistically significant.

## Results

### Recombinant protein expression and structural analysis

**Figure 2:**
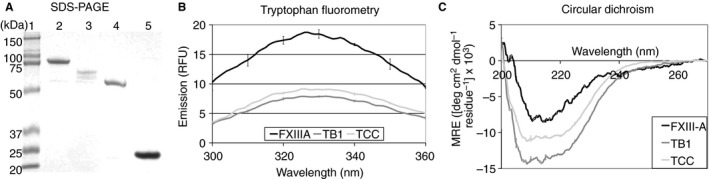
Structure of Factor XIIIA fragments. The size and purity of each fragment was confirmed by SDS‐PAGE of recombinant proteins (A). Lane 1 – molecular weight markers; lane 2 – full length rFXIIIA; lane 3 – TB1 (lacking barrel 2); lane 4 – TCC (lacking barrels 1 and 2); lane 5 – TBS (lacking barrels 1 and 2, and catalytic core). The tertiary and secondary structure of the full length rFXIIIA and fragments TB1 and TCC was determined using tryptophan fluorometry (B) and circular dichroism (C). Results shown are the average ± SD of triplicate scans. FXIIIA, full length FXIII A subunit; TB1, full length FXIII A subunit lacking barrel 2; TBS, full length FXIII A subunit lacking barrels 1 and 2, and catalytic core; TCC, full length FXIII A subunit lacking barrels 1 and 2.

Recombinant full‐length FXIII‐A, TB1, TCC and TBS (Fig. 1B–D) were successfully expressed and purified (Fig. 2A). Full‐length recombinant FXIII‐A has a *λ*_max_ of 328 nm, corresponding to predominantly buried tryptophan side chains, in agreement with the crystal structure (Fig. 2B; 1GGU 34). Recombinant TB1 and TCC have reduced fluorescence yields (areas under spectra) as a result of the loss of three tryptophan residues located within β‐barrel 2. A *λ*_max_ of 329 nm was observed with full‐length FXIII‐A, TB1, and TCC, indicating that the TB1 and TCC tryptophans are maintained in a buried environment. As these truncation variants have identical tryptophan locations, within the catalytic core and β‐sandwich, the similarities in *λ*_max_ and fluorescence yield suggest that their folding is highly comparable, and thus that the enzymatically important catalytic core retains its tertiary structure in both proteins.

CD spectroscopy was used to examine the secondary structure of full‐length recombinant FXIII‐A, TB1, and TCC. The CD spectra of recombinant FXIII‐A has a negative CD signal at 215 nm, indicative of ordered secondary structure 35, 36 (Fig. 2C). Both truncation variants have increased CD signals and double minima at 208 nm and 220 nm, characteristic of an increased percentage of α‐helical content, consistent with loss of β‐barrels 1 and 2 35, 36, 37. In conjunction, fluorescence and CD spectroscopy indicated that removal of β‐barrels 1 and 2 does not cause loss of secondary or tertiary structure in the remaining protein.

### Recombinant protein activity

**Figure 3:**
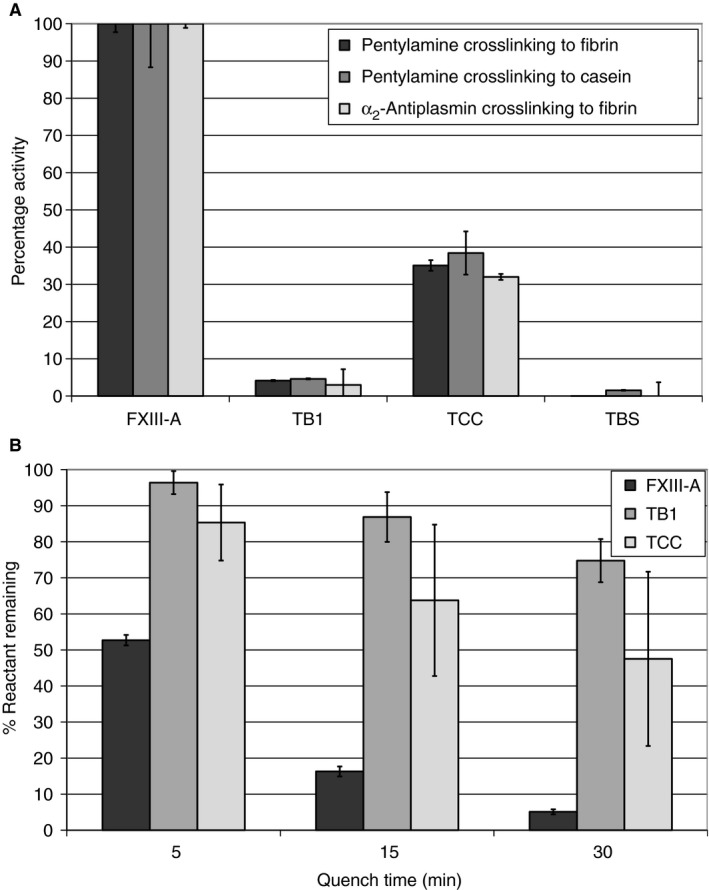
Factor XIIIA activity assays. The activity of the full length rFXIIIA and fragments TB1 (lacking barrel 2), TCC (lacking barrels 1 and 2), and TBS (lacking catalytic core and barrels 1 and 2) was investigated by their ability to incorporate either a2‐antiplasmin into plates coated with fibrinogen or 5‐(Biotinamido) pentylamine into plates coated with fibrin or casein (A). Additionally a smaller, 9 amino acid substrate was used to determine relative activity of each of the fragments (B). Results shown are mean values as a percentage of full length rFXIIIA ± SD (*n* = 3). FXIIIA, full length FXIII A subunit; TB1, full length FXIII A subunit lacking barrel 2; TBS, full length FXIII A subunit lacking barrels 1 and 2, and catalytic core; TCC, full length FXIII A subunit lacking barrels 1 and 2.

The activities of recombinant FXIII‐A, TB1, TCC and TBS were determined according to their ability to incorporate either α_2_‐antiplasmin into plates coated with fibrinogen, or 5‐(biotinamido)pentylamine into plates coated with fibrinogen or casein (Fig. 3A). TCC (shortened to residue 513) also showed activity, although this was reduced to ~ 30% of that of full‐length FXIII‐A. TB1, in which β‐barrel 2 is eliminated, showed very little activity, whereas TBS showed no activity, owing to the absence of the catalytic core domain.

### Determination of transglutaminase activity with a Q‐containing substrate peptide

The MALDI‐TOF MS‐based assay was used to assess whether thrombin‐activated recombinant FXIII‐A, TB1 and TCC could covalently crosslink the lysine mimic GEE to the glutamine‐containing K9 peptide (Fig. 3B). Over the course of the assay, the MALDI‐TOF MS peak for the K9 peptide (954 *m*/*z*) decreased in intensity over time, whereas that for the K9 peptide–GEE product (1040 *m*/*z*) increased. The percentage of reactant remaining was then calculated. As shown in Fig. 3B, 53% ± 2% of K9 substrate remained after 5 min of reaction with recombinant FXIII‐A. By 30 min, only 5% ± 0.7% of free K9 peptide remained. TB1 and TCC, missing one or both β‐barrels, could still recognize and catalyze crosslinking reactions at the active site, although with reduced activity as compared with full‐length FXIII‐A. The results further demonstrate that the catalytic cores of TB1 and TCC are able to accommodate a Q‐containing substrate peptide of 10 amino acids.

### Clot polymerization

**Figure 4:**
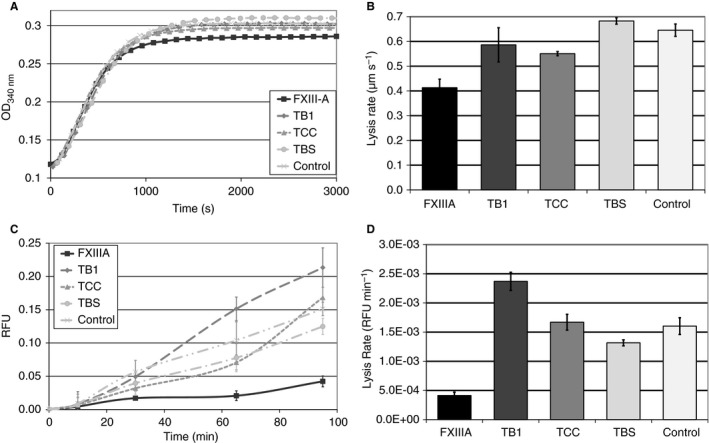
Effect of recombinant Factor XIIIA and fragments on turbidity and clot lysis. Polymerisation of FXIIIA depleted fibrinogen was initiated by the addition of thrombin, CaCl2, and either full length rFXIIIA (black square), fragment TB1 (lacking barrel 2; dark grey diamond), TCC (lacking barrels 1 and 2; mid‐grey triangle), TBS (lacking catalytic core and barrels 1 and 2; light grey circles), or a control (buffer; very light grey crosses). Generation of turbidity was measured every 12 s for 60 min and results shown are mean values of triplicate experiments (A). Only clots with full‐length FXIII‐A had a significant decrease in final maximum absorbance compared to control (*n* = 3, *P* < 0.05). The truncations did not show any significant difference from the control (*n* = 3, *P* > 0.05). Clots were also formed in either uncoated Ibidi μ‐Slides for studies with confocal microscopy (B) or in a chandler loop system (C–D) using the same conditions as for the turbidity experiments. Lysis was initiated by the addition of plasminogen and tPA. In confocal microscopy (B), the clot was visualised under a low magnification every 20 s and the time taken for the lysis front to migrate from a fixed point was measured. Lysis front velocity was determined and used to calculate the mean overall lysis rate in μm/sec ± SEM of triplicate clots. In the chandler loop system (C–D), samples of the supernatant were taken every 30 min and the released fluorescence was measured at excitation of 494 nm and emission of 519 nm to determine average relative fluorescent units (RFU) for each time point ± SEM of triplicate experiments. For both confocal microscopy and Chandler loop methods, only clots formed with full‐length FXIII‐A had a significant decrease in fibrinolysis rate compared to control (*n* = 3, *P* < 0.05). The truncations did not show any significant difference from the control in either method (*n* = 3, *P* > 0.05). FXIIIA, full length FXIII A subunit; TB1, full length FXIII A subunit lacking barrel 2; TBS, full length FXIII A subunit lacking barrels 1 and 2, and catalytic core; TCC, full length FXIII A subunit lacking barrels 1 and 2.

The effects of recombinant FXIII‐A, TB1, TCC, TBS and control (buffer only) on clot fiber thickness were investigated with the turbidity technique. Only clots with full‐length FXIII‐A showed a significant decrease in final maximum absorbance as compared with control (*n* = 3, *P* < 0.05). The final turbidity for the truncation variants was not significantly different from that for the control (*n* = 3, *P* > 0.05; Fig. 4A).

### Clot lysis

Lysis rates of clots formed in the presence of recombinant FXIII‐A, TB1, TCC, TBS or control were investigated with a static confocal microscopy method and under flow in the Chandler loop. After the addition of fibrinolytic agents to the clot, we observed a significant decrease in the rate of fibrinolysis only for clots formed in the presence of FXIII‐A as compared with control in both the confocal microscopy (*n* = 3, *P* < 0.05; Fig. 4B) and the Chandler loop experiments (*n* = 3, *P* < 0.05; Fig. 4C,D). None of the truncation variants showed a significant decrease in fibrinolysis rate with either method (*n* = 3, *P* > 0.05; Fig. 4B–D).

### Crosslinking of fibrin

**Figure 5:**
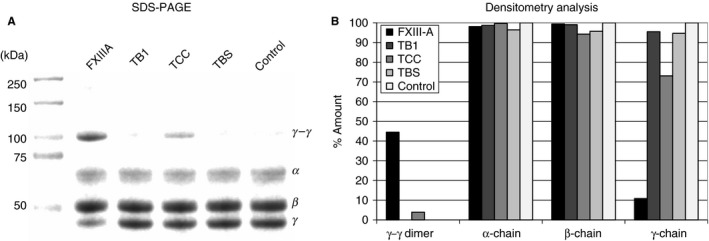
Cross‐linking of fibrin by full fength rFXIIIA and fragments. Clots were formed with fibrinogen, thrombin, CaCl2, and either full length rFXIIIA (lane 2), fragment TB1 (lacking barrel 2; lane 3), TCC (lacking barrels 1 and 2; lane 4), TBS (lacking catalytic core and barrels 1 and 2; lane 5), or a control without any form of FXIII (lane 6). After 30 min, samples were run on an SDS‐PAGE gel under reducing conditions (A). The mean density of the different bands was determined relative to the control for that peptide chain or cross‐linked structure (B). FXIIIA, full length FXIII A subunit; TB1, full length FXIII A subunit lacking barrel 2; TBS, full length FXIII A subunit lacking barrels 1 and 2, and catalytic core; TCC, full length FXIII A subunit lacking barrels 1 and 2.

The formation of fibrin clots over time in the presence of recombinant FXIII‐A, TB1, TCC, TBS or control was investigated on 4–12% Bis–Tris SDS‐PAGE gels under reducing conditions to determine the degree of α‐chain and γ‐chain crosslinking. After 30 min, 90% of the γ‐chain had been incorporated into the clot formed with full‐length FXIII‐A, and ~ 25% of the γ‐chain had been incorporated into the clot formed with TCC, all forming γ–γ dimers. The amount of unconverted γ‐chain remained at 100% in the clots formed with TB1 and TBS, relative to the control clot formed without any FXIII (Fig. 5A,B).

## Discussion

Efficient activation of FXIII is essential for the development of a mechanically stable fibrin clot that is resistant to lysis. The majority of this fibrinolysis resistance conferred by FXIII is a result of the crosslinking of α_2_‐antiplasmin to fibrin 38. However, the effect of FXIII on the fibrin structure itself may also contribute in part to increased resistance to fibrinolysis 32. Factors involved in the regulation of FXIII activation include thrombin, calcium, and fibrin 12, 15, 18, 39, 40, 41. However, the function of each individual domain of the A‐subunit with respect to FXIII activity has hitherto been undetermined. In this study, we investigated the role of the two β‐barrel domains in FXIII activity.

Recombinant full‐length FXIII‐A, TCC (both β‐barrels removed) and TB1 (β‐barrel 2 removed) all retained secondary and tertiary structure; thus, the β‐barrels are not required to maintain the overall, global conformations of the catalytic core and the β‐sandwich domain. Furthermore, the four FXIII domains have been reported to be independent folding units 42. The assays performed showed that removal of β‐barrel 2 (TB1) leads to almost total loss of transglutaminase activity, whereas the additional removal of β‐barrel 1 (TCC) returns the enzymatic activity to ~ 30% of that of activated full‐length FXIII‐A. This effect is observed not only with large, more physiological substrates (fibrin, casein, and α_2_‐antiplasmin), but also with smaller model substrates (K9 peptide and [biotinamido]pentylamine). Moreover, these transglutaminase assays made it possible to monitor the reactive glutamine and the reactive lysine residues crosslinked by activated FXIII‐A. It was also important to consider the influences on clot formation and clot lysis. Full‐length FXIII‐A remained the best at supporting clot polymerization and reducing clot lysis. Similar to the 30% enzymatic activity mentioned above, TCC catalyzed fibrin γ–γ formation with a reactivity that was 25% of wild‐type FXIII. Little to no fibrin γ–γ formation occurred with TB1 and TBS.

FXIII contains a secondary thrombin cleavage site at the K513–S514 peptide bond located within the C‐terminal portion of the catalytic domain 43, 44. The 51‐kDa protein that results from this cleavage has been reported to still bind fibrin and show transglutaminase activity despite lacking the two β‐barrels 44, 45. Interestingly, fibrin crosslinking was reduced to ~ 30% of that obtained with activated full‐length FXIII‐A, consistent with the results shown here for recombinant TCC. Lai *et al*. proposed that the truncated FXIII could no longer promote effective binding and alignment of the reactive Q and K substrates 45. The data on TB1 versus TCC presented in this article suggest, for the first time, that the β‐barrel 2 domain plays an important role in maintaining the proper conformational environment for the transglutaminase reaction. Without the structural support of β‐barrel 2, β‐barrel 1 hinders actions within the FXIII‐A catalytic core domain. Prior studies have indicated that the 19‐kDa proteolytic product (residues 514–731, β‐barrels 1 and 2) is unable to bind fibrin 44. This result demonstrates that the two β‐barrel domains exert their influences as part of the full FXIII‐A molecule, and not through the supporting fibrin scaffold 44.

Solvent accessibility studies involving amide proton hydrogen–deuterium exchange (HDX) have revealed that both FXIII β‐barrel domains participate in conformational changes occurring during both proteolytic (thrombin with calcium) and non‐proteolytic (calcium only) activation of FXIII 13, 16, 23. The β‐barrels of transglutaminase 2 have also been shown via HDX studies to undergo similar alterations upon calcium‐dependent enzyme activation 46. Members of the transglutaminase family all have a tyrosine residue (FXIII, Y560; TG2, Y516; and TG3, Y525) whose hydroxyl group is hydrogen‐bonded to the thiolate group on the active site cysteine 24. As part of the activation process, this tyrosine must be displaced from the active site region, and it has been proposed that movements of both β‐barrels promote this conformational change 24, 47. Studies involving HDX coupled with MS have revealed that the FXIII‐A β‐barrel 1 segments 533–551, 556–559 and 560–573 48, 49 become more exposed to solvent upon activation in the presence of increasing concentrations of calcium. The increased exposure of FXIIII‐A residues 533–573 is consistent with this region of β‐barrel 1 no longer undergoing close interactions with the catalytic core surface. This FXIII‐A segment may thus aid in displacing Y560 from the FXIII‐A active site. In response, the β‐barrel 2 domain could serve as a lever to help direct β‐barrel 1 away from the catalytic core domain of FXIII.

Without this β‐barrel 2 lever action, there may be greater difficulties in exposing the active site C314, leading to almost no enzymatic activity. Such a loss was observed in this study with TB1, which contains β‐barrel 1 but not β‐barrel 2. With TB1, crosslinking reactions involving fibrin, casein, α_2_‐antiplasmin and model substrates are all greatly hindered. Furthermore, the transglutaminase effects on the rates of clot formation and clot lysis are lost. The additional removal of β‐barrel 1 then causes enzymatic activity to return to 30% of that of full‐length FXIII‐A. Once again, the different glutamine‐containing and lysine‐containing substrates can better access the catalytic core regions involved in the transglutaminase reaction. An unresolved question is why TCC, lacking both β‐barrels, shows reduced activity relative to wild‐type FXIII‐A. The β‐barrels may play a protective role in the zymogen form of FXIII‐A. Once the β‐barrels are lost, transglutaminase activity is possible, but the catalytic core may become more vulnerable to biochemical attack at the active site or surrounding regions.

The first crystal structure of FXIII‐A_2_ trapped in an active conformation by a bound ligand was recently published by Stieler *et al*. 50. Ac‐Asp‐Michael acceptor (MA)‐Nle‐Nle‐Leu‐Pro‐Trp‐Pro‐OH (ZED1301) was used as the inhibitory peptide to target FXIII. The MA group serves as a glutamine side chain analog that attaches covalently to the catalytic C314. FXIII‐A_2_ was non‐proteolytically activated with calcium and exposed to ZED1301 50. The resultant crystal structure showed the FXIII‐A_2_ dissociated into two monomeric A‐subunits. The FXIII β‐barrel 1 and 2 domains rotated away from the catalytic core region, and were directed upwards towards the β‐sandwich domain. The exposed FXIIIa active site region containing the bound peptide could be viewed for the first time. This X‐ray crystal structure supports the proposed models for how TB1 and TCC work. Without the β‐barrel 2 lever, there may be difficulties in moving β‐barrel 1 into its correct position to help expose the FXIII catalytic site region.

The FXIII truncated variants highlight the roles of the individual β‐barrel domains found within FXIII‐A. The results of this study suggest that β‐barrels 1 and 2 are not required to maintain the overall, global conformation of the catalytic core domain. These two β‐barrel domains are, however, hypothesized to have influence on the active site region. In the zymogen state, the β‐barrel 1 domain is proposed to protect the FXIII‐A active site cysteine and surrounding residues. Later, the β‐barrel 2 domain serves as a lever to help move β‐barrel 1 away and expose the active site. TB1 is therefore proposed to be such a poor transglutaminase because its β‐barrel 1 domain can no longer take advantage of the lever action provided by the β‐barrel 2 domain. Transglutaminase activity is regained with TCC, a mutant lacking both β‐barrel 1 and β‐barrel 2. This study demonstrates that the individual β‐barrels play a critical role in regulating substrate access to the FXIII active site region.

## Addendum

E. L. Hethershaw participated in study design, performed the majority of the experiments, analyzed the data, and co‐wrote the manuscript. P. J. Adamson and W. N. Goldsberry performed some experiments and reviewed the manuscript. K. A. Smith, R. J. Pease, P. J. Grant, R. A. S. Ariens, and S. E. Radford participated in study design and interpretation, and reviewed the manuscript. M. C. Maurer aided in data analysis and interpretation, and co‐wrote the manuscript. H. Philippou participated in study design and data interpretation, and co‐wrote the manuscript.

## Disclosure of Conflict of Interests

The authors state that they have no conflict of interest.

## Supporting information


**Data S1.** Methods.
