# Supplementary material for: The role of β‐barrels 1 and 2 in the enzymatic activity of factor XIII A‐subunit
Source: J Thromb Haemost. 2018 May 27;16(7):1391–401. doi: 10.1111/jth.14128 (PMC6175083; doi:10.1111/jth.14128)
Supplement: Supplementary file 1 — Data S1. Methods. [file JTH-16-1391-s001.docx]

**The role of barrels 1 and 2 in the enzymatic activity of factor XIII-A**

**Supplementary Material**

**Data S1. Methods**

*Production of recombinant FXIII A subunit*

Recombinant FXIII A subunit (FXIIIA) was produced as described previously(1). Briefly, full length recombinant FXIIIA was PCR amplified from pGF13A2 (a kind gift of Prof. Charles S. Greenberg, Duke University Medical Centre, North Carolina, USA(2)) in a reaction containing 200 ng template, 500 nM 5’ and 3’ primers (forward primer – ATCGAAGCTCGTGGGAGATCTGCGGCCATGGCAGAAACT with Bgl II site underlined and reverse primer – GCAAGACTACAAGCGGCCGCATGCCAGGGT*TCA*TCT with Not I site underlined and stop codon in italics), 125 nM dNTPs, 1.5 mM MgCl_2_ and 2.6 U Expand High Fidelity enzyme mix (Roche Diagnostics, Basel, Switzerland) using the manufacturers recommended cycling conditions. Amplified product was restriction digested with Bgl II (all enzymes from New England Biolabs, Ipswich, MA, USA) and Not I and cloned into the Bam HI (overhanging fragment compatible for Bgl II but destroys cloning site) and Not I sites of pGEX-6P-1 (GE Healthcare, Chalfont St. Giles, UK) at a ratio of 3:1 using 10 U T4 DNA Ligase (Promega, Madison, WI, USA) following the manufacturer’s instructions. The construct was transformed into DH5α *E. coli* (Invitrogen) and colonies screened for the presence of insert by restriction digestion with Eco RV and Xho I.

*Production of recombinant FXIIIA truncations*

Truncations of recombinant FXIIIA (Figure 1), including truncations to residue 628 [truncated to barrel 1 (TB1)], 515 [truncated to the catalytic core (TCC)] and 184 [truncated to beta sandwich (TBS)], were PCR amplified from the full length recombinant FXIIIA construct (FXIIIA-pGEX-6P-1). The same 5’ primer for each truncation and the 3’ primer that shortened the recombinant FXIIIA to the end of the appropriate truncation were employed with the incorporation of Bam HI and Xho I sites, in a similar manner as used for the full length construct. The inserts were ligated into the Bam HI and Xho I sites of pGEX-6P-1 and the resulting constructs transformed into DH5α *E. coli*. Colonies for each construct were screened for the presence of the inserts using restriction digestion analysis with Xho I and Eco RV. All recombinant FXIIIA constructs, including the full length FXIIIA, were sequenced using the BigDye Terminator v3.1 Cycle Sequencing Kit (Applied Biosystems, Foster City, CA, USA) on an ABI Prism 310 Genetic Analyser (Applied Biosystems) to ensure the integrity of the PCR cloning.

*Expression and purification of recombinant proteins*

Unless stated otherwise, all reagents were from Fisher Scientific (Loughborough, UK). All constructs were transformed into BL21 Gold (DE3) *E. coli* (Stratagene, La Jolla, CA, USA) for expression. All recombinant proteins were expressed and purified as previously described for the αC domain of fibrinogen and recombinant FXIIIA(1). Proteins were concentrated up to the required concentration using Aquacide II (Merck Chemicals Ltd, Nottingham, UK) and dialysed against PBS with 5% glycerol for 6 hours, changing the buffer after 3 hours. The recombinant proteins were analysed by SDS-PAGE, fluorometry, circular dichroism, and FXIII activity assays.

References

(1) Smith KA, Adamson PJ, Pease RJ, Brown JM, Balmforth AJ, Cordell PA, Ariëns RA, Philippou H, Grant PJ. Interactions between factor XIII and the alphaC region of fibrinogen. Blood 2011;117:3460-8.

(2) Lai TS, Santiago MA, Achyuthan KE, Greenberg CS. Purification and characterization of recombinant human coagulant factor XIII A-chains expressed in E. coli. Protein Expr Purif 1994;5:125-32.
